# Prognostic Impact of Acute Cardiovascular Events in COVID-19 Hospitalized Patients—Results from the CORONA Germany Study

**DOI:** 10.3390/jcm10173982

**Published:** 2021-09-02

**Authors:** Melanie A. Gunawardene, Nele Gessler, Peter Wohlmuth, Kathrin Heitmann, Philipp Anders, Kai Jaquet, Christoph U. Herborn, Dirk Arnold, Berthold Bein, Martin W. Bergmann, Klaus R. Herrlinger, Axel Stang, Ruediger Schreiber, Claas Wesseler, Stephan Willems

**Affiliations:** 1 Department of Cardiology and Internal Intensive Care Medicine, Asklepios Hospital St. Georg, 20099 Hamburg, Germany; n.gessler@asklepios.com (N.G.); s.willems@asklepios.com (S.W.); 2 Asklepios Proresearch, Research Institute, 20099 Hamburg, Germany; p.wohlmuth@asklepios.com (P.W.); ka.heitmann@asklepios.com (K.H.); k.jaquet@asklepios.com (K.J.); c.herborn@asklepios.com (C.U.H.); 3 Faculty of Medicine, Semmelweis University, 1085 Budapest, Hungary; philipp.anders@semmelweis-hamburg.de (P.A.); a.stand@asklepios.com (A.S.); 4 Asklepios Hospitals GmbH & Co. KGaA, 20099 Hamburg, Germany; 5 Department of Hematology, Oncology, Palliative Care Medicine and Rheumatology, Asklepios Hospital Altona, 22763 Hamburg, Germany; d.arnold@asklepios.com; 6 Department of Anesthesiology and Intensive Care Medicine, Asklepios Hospital St. Georg, 20099 Hamburg, Germany; b.bein@asklepios.com; 7 Department of Internal Medicine—Cardiology and Pneumology, Asklepios Hospital Wandsbek, 22043 Hamburg, Germany; mar.bergmann@asklepios.com; 8 Department of Internal Medicine I—Gastroenterology, Oncology, Infectious Diseases, Asklepios Hospital Nord-Heidberg, 22417 Hamburg, Germany; k.herrlinger@asklepios.com; 9 Department of Oncology and Palliative Care Medicine, Asklepios Hospital Barmbek, 22307 Hamburg, Germany; 10 Department of Anesthesiology and Intensive Care Medicine, Asklepios Hospital West, 22559 Hamburg, Germany; r.schreiber@asklepios.com; 11 Department of Pneumology, Asklepios Hospital Harburg, 21075 Hamburg, Germany; c.wesseler@asklepios.com

**Keywords:** atrial fibrillation, troponin, cardiovascular disease, coronavirus, COVID-19

## Abstract

Background: Acute myocardial injury (AMJ), assessed by elevated levels of cardiac troponin, is associated with fatal outcome in coronavirus disease 2019 (COVID-19). However, the role of acute cardiovascular (CV) events defined by clinical manifestation rather than sole elevations of biomarkers is unclear in hospitalized COVID-19 patients. Objective: The aim of this study was to investigate acute clinically manifest CV events in hospitalized COVID-19 patients. Methods: From 1 March 2020 to 5 January 2021, we conducted a multicenter, prospective, epidemiological cohort study at six hospitals from Hamburg, Germany (a portion of the state-wide 45-center CORONA Germany cohort study) enrolling all hospitalized COVID-19 patients. Primary endpoint was occurrence of a clinically manifest CV-event. Results: In total, 132 CV-events occurred in 92 of 414 (22.2%) patients in the Hamburg-cohort: cardiogenic shock in 10 (2.4%), cardiopulmonary resuscitation in 12 (2.9%), acute coronary syndrome in 11 (2.7%), de-novo arrhythmia in 31 (7.5%), acute heart-failure in 43 (10.3%), myocarditis in 2 (0.5%), pulmonary-embolism in 11 (2.7%), thrombosis in 9 (2.2%) and stroke in 3 (0.7%). In the Hamburg-cohort, mortality was 46% (42/92) for patients with a CV-event and 33% (27/83) for patients with only AMJ without CV-event (OR 1.7, CI: (0.94–3.2), *p* = 0.077). Mortality was higher in patients with CV-events (Odds ratio(OR): 4.8, 95%-confidence-interval(CI): [2.9–8]). Age (OR 1.1, CI: (0.66–1.86)), atrial fibrillation (AF) on baseline-ECG (OR 3.4, CI: (1.74–6.8)), systolic blood-pressure (OR 0.7, CI: (0.53–0.96)), potassium (OR 1.3, CI: (0.99–1.73)) and C-reactive-protein (1.4, CI (1.04–1.76)) were associated with CV-events. Conclusion: Hospitalized COVID-19 patients with clinical manifestation of acute cardiovascular events show an almost five-fold increased mortality. In this regard, the emergence of arrhythmias is a major determinant.

## 1. Introduction

Acute myocardial injury is associated with a poor outcome in coronavirus disease (COVID-19) [1]. In most publications, acute myocardial injury is defined by the Fourth Universal Definition of Myocardial Infarction naming an increase of cardiac troponin levels with at least one value above the 99th percentile upper reference limit [1,2]. It is considered acute, if a dynamic in troponin values is observed [2]. Irrespective of the current pandemic, acute myocardial injury without signs or symptoms indicating overt cardiac ischemia is associated with a worse prognosis [3]. Also, the presence of preexisting cardiovascular disease has been reported to worsen overall outcome in hospitalized COVID-19 patients [4].

Further, a detailed characterization on clinical manifestations of acute cardiovascular events that are not only defined by sole elevations of biomarkers are limited in patients being hospitalized with COVID-19. In this study, we investigated hospitalized COVID-19 patients with acute clinically manifest cardiovascular events.

## 2. Methods

### 2.1. Study Design

**Figure 1:**
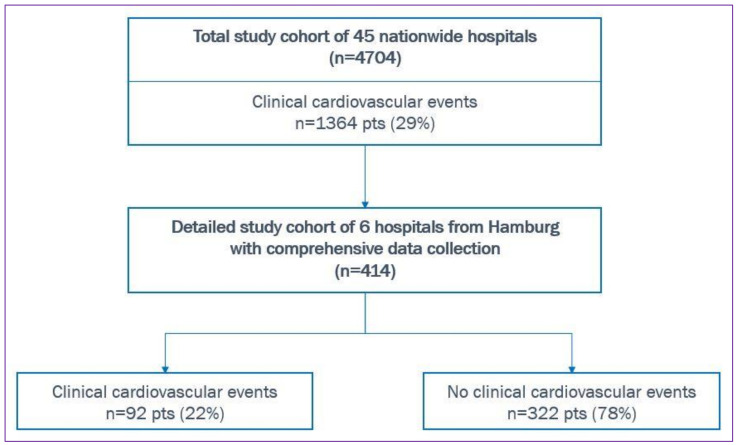
Flow chart of enrollment, cohorts and cardiovascular events. pts, patients.

In this manuscript, we report results of the Hamburg cohort as part of the “CORONA Germany” study, including six hospitals from Hamburg specializing in cardiovascular care (Figure 1). The “CORONA Germany”—*Clinical Outcome and Risk in hospitalized COVID-19 patients*—study (*ClinicalTrials.gov*, Trial registration number: *NCT04659187*) is a multicenter observational, prospective, epidemiological cohort study. It was conducted in 45 hospitals across Germany that were all part of the same hospital network. The trial was investigator-initiated; the steering committee was responsible for design, execution, and conduct of the study. The study was approved by the institutional review board and ethics committee. It was performed according to the principles of the Declaration of Helsinki. There was no funding, the study was fully investigator-initiated. All members of the steering committee approved the predefined statistical analyses and interpretation of the data. The decision to publish the results and decisions regarding the contents of the manuscript were made by the steering committee. The authors attest to the accuracy of the data and of all analyses. All data collected from the data warehouse were compared to and validated by the networks’ quality management data base. All endpoints were reviewed by an endpoint committee provided by the networks research institute and medical trained staff. Patients’ charts were reviewed.

The key inclusion criterion was enrollment of all consecutive hospitalized patients tested positive for SARS CoV-2 using a reliable test method. Patients with negative SARS CoV-2 testing were excluded from this study.

Data of these COVID-19 patients were entered in a database as explained in the following: first, patients from all network centers in Germany were collected and included. Data were extracted from the network’s data warehouse. From this source, patients were extracted anonymously to the study data base for the Hamburg cohort analysis (consisting of six centers from Hamburg, Germany) and daily assessment of these patients was performed. Due to the current pandemic situation of SARS CoV-2 the total sample size was unknown when the study was started.

### 2.2. Study Cohort

Patients were enrolled from 1 March 2020 until 5 January 2021. The study included all patients coded with international statistical classification of diseases and related health (ICD) for coronavirus SARS CoV-2 infection and need for hospitalization. Data about age, gender, death, need for intensive medical care, mechanical ventilation, and cardiovascular events were given. In the Hamburg cohort, each individual of the study population was monitored during the whole length of the hospital stay. Data entry was performed in a structured way, anonymously. The data fields were pre-formatted and periodic data checks ensured a high data quality. This dataset included overall 95 parameters of baseline symptoms, baseline vital parameters, baseline laboratory findings, prior medication, preexisting comorbidities, and electrocardiogram (ECG) findings (details on the variables are shown in the Supplementary Materials S1). Cardiac high-sensitivity troponin I was measured by the *ARCHITECT* assay (©Abbott, Abbott Park, IL, USA) with a gender specific 99th percentile upper limit reference of 16 ng/L in females and 34 ng/L in males. Patients with solely acute myocardial injury were defined as patients with an increase of cardiac high-sensitivity troponin I above the 99th percentile upper reference limit but without occurrence of a clinical cardiovascular event.

### 2.3. Endpoints

The primary endpoint was occurrence of a clinically manifest acute cardiovascular event in any hospitalized COVID-19 patient. A cardiovascular event was defined as any one of the following clinical manifestations: (1) cardiopulmonary resuscitation in cardiac arrest, (2) cardiogenic shock, (3) acute coronary artery syndrome, including ST-segment elevation myocardial infarction (STEMI) and non-ST-segment elevation myocardial infarction (NSTEMI), (4) acute myocarditis, (5) de novo cardiac arrhythmia, (6) arterial or venous thrombosis, (7) pulmonary artery embolism, (8) worsening of prior or new onset heart failure, (9) ischemic stroke. Definitions of each clinical diagnosis can be found in the Supplementary Materials (S2).

The secondary endpoints were (1) in-clinic mortality and (2) the development of a risk stratification score to predict the primary endpoint in hospitalized COVID-19 patients in the Hamburg cohort.

### 2.4. Statistics

Demographic data, symptoms, vital data, laboratory data, cardiac marker, blood gas analysis, comorbidities, medication, and ECG data, documented at baseline, were summarized descriptively. Continuous data were shown as means +/− standard deviations and medians [25th and 75th percentiles]. Categorical data were presented as proportions and frequencies (data presented in Table 1, Table 2 and Table 3).

Based on expert-opinion and the literature with regard to predictors on COVID-19 events, 44 baseline data were taken into account. Immune-suppressive medication was aggregated to one variable. Furthermore, variables affected by informative missing and exhibiting ≥30% missing values were excluded; 25 variables, 12 continuous/discrete and 13 binary variables were used for model building (gender, age, fever, dyspnea, cough, respiratory rate, oxygen saturation, heart rate, systolic blood pressure, creatinine, CRP, LDH, aPTT, potassium, leukocytes, number of comorbidities, tumor disease, antiplatelet medication, renin-angiotensin-aldosterone antagonists, antidiabetic medication, immuno-suppressive medication, oral anticoagulation, statins, diuretics, proton pump inhibitor). The missing data were imputed using additive regression models.

A full logistic regression model using a penalized estimation approach (ridge regression) was applied. Continuous variables were transformed using restricted cubic splines with three knots (10th, 50th and 90th percentile). A fast backward method was used on a linear model regressing all 25 variables on the predictions of the logistic model. As the full model shows R2 = 1, model reduction can be applied to any degree. The final regression model was chosen to comprise a high R2 and an optimal value of the Akaike information criteria.

Resampling validation of the logistic regression model was performed on B = 200 bootstrap samples. The estimated bias due to overfitting in the final model was examined using explained variation R2 and Sommers’ D rank-correlation. Validation results are not shown.

The final regression model was presented with parameter estimates, standard errors, Wald Z statistics and *p*-values. Effects on cardio-vascular events were shown with odds ratios and 95% confidence intervals and were summarized in a forest plot style. Continuous predictors were presented as interquartile-range odds ratios (upper quartile: lower quartile) and categorical predictors as simple odds ratios (current category: reference category). Partial effects in the prediction model for the cause of a cardiovascular event were shown on a log-odds scale. A nomogram was derived from the prediction model to calculate the probability for a cardiovascular event. Event prediction in the nomogram is related on a total point score that summarizes individual points (0–100) assigned from the value of each predictor.

Baseline and maximum troponin values during hospital stay were associated to mortality in a non-adjusted manner using a logistic regression model with restricted cubic splines with three knots. The probability of event occurrence was shown with point estimates and 95% confidence intervals. Mortality rates conditional on the occurrence of cardio-vascular events are presented as line plots for the Hamburg cohort and the entire data.

All analyses except the descriptive analyses of the whole cohort (Table 1) and mortality rates from cardio-vascular events are based on the Hamburg registry. *p*-values are largely omitted. The few of them used, should be understood as two-sided with a significance level of 5%. All analyses are performed with R (R Core Team 2020), (see Supplementary Materials for statistical literature S3).

## 3. Results—Hamburg Cohort

### 3.1. Baseline Characteristics

The baseline characteristics of the 414 patients are shown in Table 1. Initial dyspnea, cough and fever were present in 219 (53%), 210 (51%) and 196 (47%) patients, respectively. The median duration between onset of initial symptoms and hospitalization was 7 [q1: 3-q3: 10] days.

At admission, baseline mean heart rate was 88 ± 19 beats per minute, systolic and diastolic blood pressure were 134 ± 22 and 77 ± 12 mmHg at a respiratory rate of 19.6 ± 6.2 breaths per minute. Baseline ECG showed sinus rhythm in 84% and atrial fibrillation in 16% of patients.

Initial laboratory findings are also shown in Table 2, including a median and mean high-sensitivity troponin of 14 (q1: 8-q3: 37) and 537 ± 7022 ng/L.

Directly after admission, the majority of patients (86%) were first transferred to a normal hospital ward and 14% to intermediate care (IMC) (2%) and/or intensive care units (ICU) (12%).

### 3.2. Cardiovascular Comorbidities and Medication

Patients comorbidities and prior medication are shown in Table 2. Cardiovascular risk factors such as diabetes mellitus, dyslipidemia and smoking were present in 103 (25%), 61 (15%) and 28 (7%) patients. Prior cardiovascular diseases were hypertension in 226 (55%), preexisting cardiomyopathy in 20 (5%), coronary artery disease in 69 (17%), a prior myocardial infarction in 29 (7%), any vascular disease in 87 (21%) and prior arrhythmias in 75 (18%) patients.

Overall, 96 (23%) patients were on oral antiplatelet therapy, 63 (15%) on oral anticoagulation. ACE-inhibitor or angiotensin-receptor blockers were used in 155 (37%), aldosterone antagonists in 23 (6%) and angiotensin-receptor-neprilysin-inhibitor (ARNI) in 2 (0.5%) patients; 124 (30%) patients received betablockers and 22 antiarrhythmic drugs (5%).

### 3.3. Clinical Results

We detected 132 cardiovascular events in 92 of 414 patients from Hamburg (22%; 1.4 events per patient) with worsening/new onset of heart failure in 43 (10.4%), de novo arrhythmia in 31 (7.4%), cardiopulmonary resuscitation in 12 (2.9%), acute coronary syndrome in 11 (2.7%), a cardiogenic shock in 10 (2.4%) patients, pulmonary embolism in 11 (2.7%), thrombosis in 9 (2.2%), ischemic stroke in 3 (0.7%) and myocarditis in 2 (0.5%) patients (Table 3, compared to the overall study population of the “CORONA Germany” cohort).

**Figure 2:**
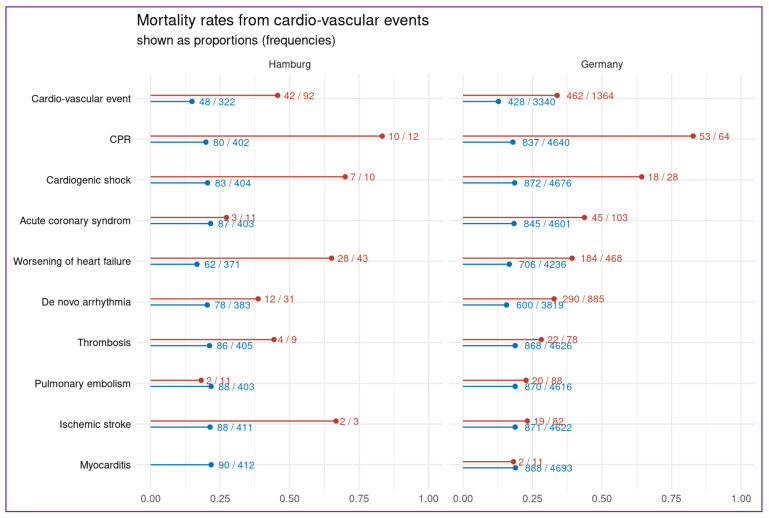
Rates of mortality in COVID-19 patients with and without a prior specific cardiovascular event. Proportion of patients dying conditional on the occurrence (red line; (“*n* (patients who died with the cardiovascular event)/*n* (all patients with that CV event“)) and non-occurrence (blue line; “*n* (patients who died without that CV event)/*n* (all patients withouth that CV event“) of specific cardiovascular events, shown on the left. The left plot displays the results of the patients from the Hamburg registry. The plot on the right is based on the entire cohort. CPR indicates cardiopulmonary resuscitation.

There were 90 deaths (*n* = 414; 22%) of whom 42 patients (46% of all deaths) had a prior cardiovascular event. In the Hamburg cohort, COVID-19 patients with de-novo cardiovascular events had a 4.8-times increased risk for death (OR 4.8, CI: (2.9–8)). Details on mortality rates for each prior cardiovascular outcome are shown in Figure 2 (including the data from the whole study cohort of the “CORONA Germany” study). Incidences of cardiovascular events and death throughout the different months of the study timespan are shown in the Supplementary Materials (S4).

When looking at the total “CORONA Germany” study cohort, a total of 4704 COVID-19 patients were hospitalized among all 45 centers (including the six centers from Hamburg; Figure 1). The primary endpoint occurred in 1364 (24%) of all COVID-19 patients, (Table 3). Death occurred in 890 of all 4704 (19%) patients. Patients with COVID-19 and a cardiovascular events had an increased risk for death (OR 3.5, CI: (3.0–4.1)), with the highest mortality rates for cardiopulmonary resuscitation (83%), cardiogenic shock (64%) and acute coronary syndrome (44%), (a listing of all mortality rates is shown in Figure 2). In total, 46 (1.0%) patients needed extracorporeal membrane oxygenation for life support.

### 3.4. Effects on Cardiovascular Outcome

**Figure 3:**
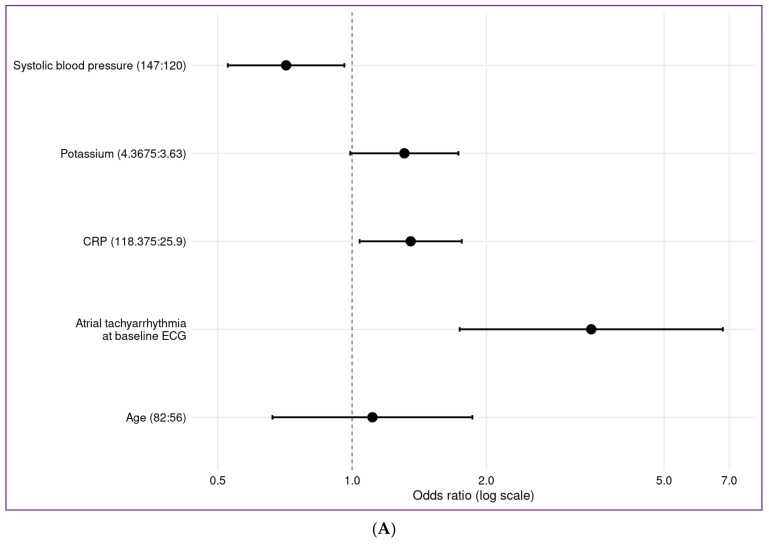

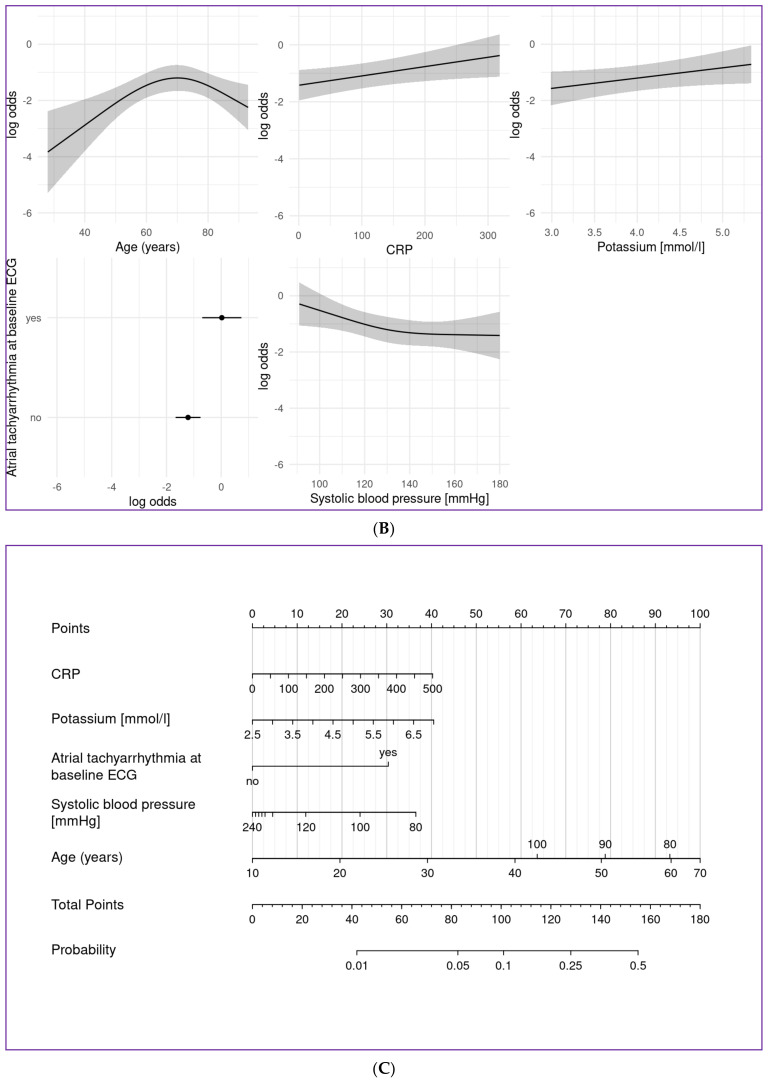
Predicting cardiovascular events from baseline data of the Hamburg cohort: (**A**) shows the interquartile-range odds ratios for continuous predictors (upper quartile: lower quartile) and simple odds ratios for categorical predictors (current category: reference category). (**B**) shows the partial effects (log-odds scale) in the prediction model for cause of a cardiovascular event. (**C**) shows the nomogram: calculating the probability for a cardio-vascular event. For each predictor, points (0–100) are assigned. The total points are associated to event prediction. CRP indicated C-reactive protein, ECG indicates electrocardiogram.

Occurrence of a clinically manifest cardiovascular event in COVID-19 hospitalized patients from Hamburg was related to age (interquartile range 82 to 56 years; OR: 1.11, CI: (0.66–1.86), presence of atrial fibrillation at the baseline ECG (OR 3.43, CI: (1.74–6.77)), baseline systolic blood pressure (interquartile range 147 to 120 mmHg, OR 0.71, CI: (0.527–0.96)), baseline potassium (interquartile range 4.4 to 3.6 mmol/L; OR 1.31, CI: (0.9911–1.73)) and C-reactive protein values (interquartile range 118 to 26 mg/L; OR 1.35, CI: (1.04–1.76) (Figure 3A,B). A nomogram derived from the regression model was used to predict the probability of cardiovascular events in COVID-19 hospitalized patients from baseline data (Figure 3C). In this prediction model, coexisting cardiovascular diseases and medication were not major indicators for the prognosis.

### 3.5. Acute Myocardial Injury and High Sensitivity Troponin

**Figure 4:**
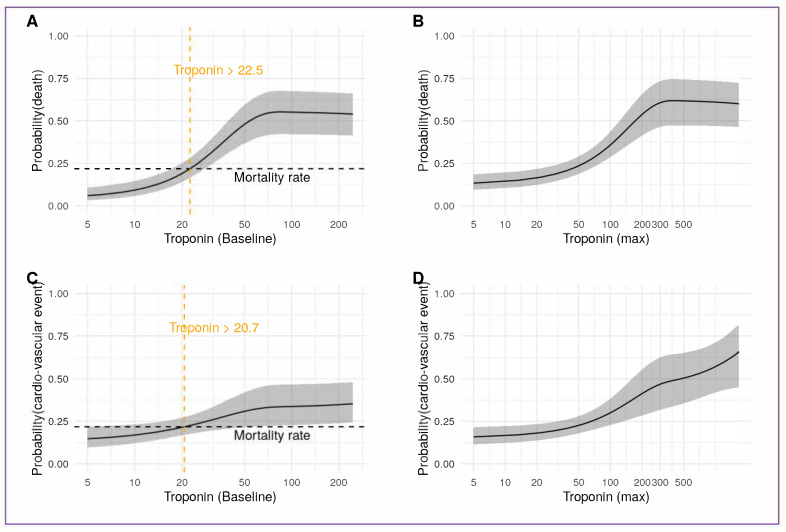
Prediction of non-surviving and cardiovascular events from high sensitivity troponin values. (**A**) shows the probability of death derived from baseline troponin values. The entire mortality rate of the Hamburg registry is shown as a horizontal dashed line. The vertical dashed line represents the lower limit of the troponin value exceeding the base mortality rate. (**B**) shows the probability of non-surviving predicted from maximum troponin values during hospital stay. (**C**) shows the probability of a cardiovascular event estimated from baseline troponin values. The mortality rate is shown as a horizontal dashed line. The vertical dashed line represents the lower limit of the troponin value exceeding the base mortality rate. (**D**) shows the probability of a cardio-vascular event predicted from maximum troponin values during hospital stay.

Risk of death was higher in patients suffering from a clinically manifest cardiovascular event as compared to patients with acute myocardial injury only (elevated troponin but no clinical event), (OR 1.7 (CI: (0.94–3.2; *p* = 0.077)) in the Hamburg cohort. The baseline and maximum high-sensitivity troponin values were both associated with increased probability for a clinically manifest cardiovascular event and death (Figure 4A–D). With a high sensitivity troponin of more than 22.5 ng/L the risk for mortality exceeded the cohorts baseline risk for death (Figure 4A).

Compared to patients with only acute myocardial injury, patients with clinically manifest cardiovascular event were younger (interquartile range 84 to 69.5 years, OR 0.54, CI: (0.37–0.81)) and presented more often with atrial fibrillation at baseline ECG (OR 2.83, (1.29–6.20)).

## 4. Discussion

In this multicenter prospective observational study, clinical manifestations of acute cardiovascular events were highly common and associated with an almost five-fold increase in risk for death in COVID-19 hospitalized patients. Severe events such as cardiopulmonary resuscitation and cardiogenic shock were frequently fatal for COVID-19 patients. Acute cardiovascular events seemed to have a higher impact on mortality than acute myocardial injury alone. Both, baseline and maximum high-sensitivity troponin levels were linked to an increased risk for clinically manifest cardiovascular events and death. Additionally, newly diagnosed arrhythmias, mostly atrial tachyarrhythmias, were a major driver on cardiovascular outcome in hospitalized COVID-19 patients.

In our study, we present a simple risk score for prediction of the primary endpoint, which encompasses older age, presence of atrial fibrillation at baseline ECG, lower baseline systolic blood pressures and higher C-reactive protein and potassium levels.

### 4.1. Cardiovascular Events and Troponin

The findings of this study are of high importance as they highlight the fact that clinically manifest cardiovascular events are not only common but impact overall survival in COVID-19 patients. Recently, the American Heart Association launched a COVID-19 cardiovascular disease registry with similar outcomes (mortality, stroke, myocardial infarction, arrhythmia, deep vein thrombosis/pulmonary embolism, length of stay) [5]. Also, the CAPACITY-COVID registry from Europe was initiated with the purpose of determining the role of cardiovascular disease in the current pandemic [6], pointing out the need for further research regarding clinical cardiovascular outcomes. In this study, we overcome certain limitations of previous studies by providing a prospective multicenter study with a larger sample size, a systematic approach with high accuracy data collection and an endpoint verification including patient-follow up throughout the complete hospital stay [1,7,8,9,10]. We observed that in about every fifth patient associated with clinical deterioration transfer to an ICU was necessary.

Furthermore, we present a combined primary endpoint consisting of purely clinical cardiovascular events in COVID-19 patients. As opposed to other studies, we did not include acute myocardial injury without clinical events (sole elevation of troponin) [1,11]. Further analysis of our results showed us that acute cardiovascular events seemed to have a higher impact on mortality than acute myocardial injury alone.

Troponin elevations alone, regardless of the presence of COVID-19 are associated with a higher risk for poor outcome [3,10]. Discrimination between clinically manifested events and sole laboratory findings are mandatory in order to treat the underlying cause correctly and to adjust the severity of risk. Another prospective study postulated that differences in baseline biomarkers among COVID-19 ICU patients and non-survivors did not persist when accounting for clinical characteristics [12]. Troponin levels can be elevated due to multiple reasons, including elevated levels in chronic kidney disease and patients with atrial fibrillation [13,14]. A sole measure of troponin does not confirm acute cardiovascular disease but can be a bystander to other severe diseases, e.g., pneumonia [15].

### 4.2. Prediction Models

In this study, the primary endpoint was more likely to occur with older age (but a decrease of risk in very old patients), presence of atrial fibrillation at baseline ECG, a lower baseline systolic blood pressure (with a plateau of risk in increased blood pressures) and with increasing values in C-reactive protein and potassium. Another predictive model for cardiovascular outcome in COVID-19 has been published, which included both, cardiovascular events and acute myocardial injury/troponin elevation [11]. However, some of the statistical approaches necessitate further discussion: the clinical outcomes were treated as groups, model selection was based on one-dimensional *p*-values and the score assigned to each predictor was arbitrary. Although model validation was performed it was based on only one simple data split. Except for age, the other nine risk factors they found were discrepant from our findings (gender, cough, chronic heart disease, lymphocyte count, blood urea nitrogen, glomerular function rate, partial thromboplastin time, d-dimer and procalcitonin). In comparison, our study reports a simple prognostic tool to assess the risk for only clinically manifested cardiovascular events in COVID-19 hospitalized patient. Another simple prognostic tool has been reported by Ruscica et al. warranting only age and three biochemical parameters (N- terminal pro- B- type natriuretic peptide, interleukin 6 and lactate dehydrogenase) to quickly assess the risk of in-hospital mortality in COVID-19 patients [16].

### 4.3. Impact of Clinical Baseline Parameters—Age

Age itself has been reported to be a risk factor for poor outcome and more severe disease in COVID-19 patients [4,11,17]. Our study confirms this finding regarding cardiovascular events. Interestingly, the risk for cardiovascular events peaks around the age of 70 and decreases afterwards. A possible hypothesis could be that patients above 70, infected with COVID-19 have a fatal outcome before a possible occurrence of another endpoint.

### 4.4. Atrial Fibrillation

The presence of atrial fibrillation at the baseline ECG was found as a prognostic indicator in our study. This is in alignment with prior findings [18]. Of note, the baseline ECG in our study was taken in every patient, regardless of symptoms or presence of prior arrhythmias to detect the baseline rhythm of each patient in the study cohort. Reasons for increased rates of arrhythmias in COVID-19 patients have been discussed extensively and include injuries to the myocardium by hypoxia, increased inflammatory responses, direct viral tissue involvement and decompensation of underlying cardiac diseases [18].

Additionally, our findings show that de novo symptomatic and asymptomatic arrhythmias (in patients without a prior history of cardiac arrhythmias) occur frequently during hospitalization and are one of the major drivers for cardiovascular events in COVID-19 patients. De novo arrhythmias in our study were most often atrial tachyarrhythmias. Thus, patients with atrial fibrillation are at increased risk for cardiovascular events and should be considered as such when being hospitalized. Consequently, we suggest that rhythm monitoring should be performed generously in these patients. Atrial fibrillation should be treated and clinical risk assessment should be performed continuously among hospitalized COVID-19 patients that are not in sinus rhythm. Early rhythm control of atrial fibrillation has shown to be effective in reducing the composite of death from cardiovascular causes, stroke, or hospitalization with worsening of heart failure or acute coronary syndrome and may also be relevant in this patient population [19]. Another important point is the immediate start of oral anticoagulation in newly diagnosed atrial fibrillation to prevent thromboembolic events [20], especially since hypercoagulable states are already associated with increased risk and poor outcome in COVID-19 patients [21].

Of note, four patients in the study cohort suffered from ventricular arrhythmias of whom two (50%) did not survive. This number of patients is too low to draw any conclusions. However, three of these four patients suffered from advanced respiratory failure due to COVID-19 and are therefore considered to be at high risk for poor outcome [22]. The one patient suffering from a STEMI and ventricular fibrillation survived after successful resuscitation and percutaneous coronary intervention demonstrating the better prognostic outcome of this entity.

### 4.5. Blood Pressure

Baseline low systolic blood pressures were associated with an increase in cardiovascular events in this study. In the era before COVID-19, a consistent finding in a post hoc analysis of multiple trials regarding hypertension therapy revealed that reducing systolic blood pressure to less than 120 mmHg increased the incidence of cardiovascular events and death [23]. Accordingly, recommendations in current guidelines target a blood pressure >120 mmHg [23]. Additionally, low blood pressures in severe diseased patients can be a sign of shock, regardless of cause [14]. Thus, as shown in our study, a systolic blood pressure of less than 120 mmHg should be considered as an alarming signal in COVID-19 patients.

Interestingly, prior cardiovascular risk factors (present in 7–55% of our study participants), cardiovascular diseases (present in 5–21%) and prior medication therapy, including renin-angiotensin-aldosterone-system-inhibitors, betablockers, diuretics, statins and antidiabetic medication (present in 19–37%) were not major predictors for a manifest cardiovascular event in our model but have been subject of discussion in the current literature [4,24]. A correspondence from the European Heart Journal, for example, supports the rationale for the use of statins, as an add-on treatment for COVID-19 patients, based on their known immunomodulatory properties [25]. The authors argue that statins have a widespread availability, an optimal tolerability profile, low costs and exert pleiotropic effects on inflammation and oxidative stress, contributing to their beneficial impact on cardiovascular diseases [25]. Use of statins could therefore improve the clinical course of patients with COVID-19, either by their immunomodulatory action or by preventing cardiovascular damage [25].

Lastly, it needs to be considered that the time span covered by this study is wide and includes different waves of COVID-19 infections, burdens on the health care system and the learning curve of this new disease. As shown by our study group, in-hospital mortality showed temporal variation during the year 2020 in the “CORONA Germany” cohort, mostly influenced by age [22]. Also, we assume seasonality of COVID-19 to be noteworthy, as discussed before [22]. Interestingly, incidences of clinically manifest cardiovascular events showed two peaks in our study coinciding with the two waves of infection in Germany.

### 4.6. Limitations

Despite high quality data collection, all data are observational and were taken during hospitalization from electronic patients’ charts. For treating physicians there was no mandatory laboratory test protocol, and therefore laboratory values were requested as needed and depended on the symptoms and clinical picture of each patient. This explains missing values at baseline in some patients. The specifics on handling of missing data can be found in our statistical methods section. However, missing data could still influence findings (e.g., by selection bias) of this purely observational study.

The presence of an endpoint review committee in our study provided highly accurate and reliable results of patients’ outcome. The study population included all hospitalized COVID-19 patients, with mild to severe disease statuses and patients were at a different status of disease when being hospitalized. This could perhaps account for a selection bias. However, hospitalization itself, is a clinical marker for severity and separates the study population from very mild courses of this disease. This study does not provide any information on the cause of cardiovascular events in COVID-19 patients or whether patients presented to the hospital due to COVID-19 related respiratory symptoms or primary due to cardiovascular symptoms. Many hypothesis regarding causes have been published and seem to be multifactorial as well as, to a certain extent, unknown [4,18,24].

## 5. Conclusions

Hospitalized COVID-19 patients with the emergence of acute clinically manifest cardiovascular events are at an almost five-fold increased risk for death. Newly diagnosed atrial fibrillation is a major driver on cardiovascular outcomes.

Age, systolic blood pressures of less than 120 mmHg, elevated CRP and potassium values and/or the presence of atrial fibrillation at baseline identify COVID-19 patients at high risk for cardiovascular events and death. Thus, we suggest that these high-risk COVID-19 patients require continuous monitoring, measurement of troponin dynamics, close clinical follow up and management during hospitalization to avert poor prognosis.

## Figures and Tables

**Table 1:** Baseline characteristics—Hamburg cohort (*n* = 414).

	*N*	*n* = 414
Age (years)		73 (56–82); 68 ± 18
Gender (female)		0.42 (171)
Duration between symptoms onset until hospitalization, prop(*n*)		7.0 (3.0–10.0); 7.4 ± 6.4
Sick contact in patient history, prop(*n*)		0.24 (98)
**First allocation of patients in the hospital:**		
normal ward, prop(*n*)		0.86 (349)
intermediate care unit, prop(*n*)		0.02 (9)
intensive care unit, prop(*n*)		0.12 (47)
Hospitalization for elective procedure, prop(*n*)		0.02 (6)
Already hospitalized, prop(*n*)		0.00 (1)
**Baseline symptoms**		
Cough, prop(*n*)		0.51 (210)
Fever (>38.5 °C), prop(*n*)		0.47 (196)
Dyspnea, prop(*n*)		0.53 (219)
Headache, prop(*n*)		0.08 (34)
Diarrhea, prop(*n*)		0.15 (62)
Expectoration, prop(*n*)		0.14 (57)
Sore throat, prop(*n*)		0.07 (29)
Loss of smell/taste, prop(*n*)		0.04 (18)
Body aches, prop(*n*)		0.16 (66)
Nausea, prop(*n*)		0.16 (66)
Chills, prop(*n*)		0.08 (33)
Body temperature		37.5 ± 1.2
Glasgow coma scale < 15 points, prop(*n*)		0.03 (12)
**Baseline vital parameters**		
Heart rate (/min)		88 ± 19
Systolic blood pressure (mmHg)		134 ± 22
Diastolic blood pressure (mmHg)		77 ± 12
Respiratory rate (/min)		19.6 ± 6.2
spO2 (%)		93.2 ± 6.2
**Baseline laboratory findings**		
Lymphocytes, /nL	219	1.0 (0.6–1.5); 1.8 ± 3.7
Leukocytes, /nL	402	7.0 (5.2–9.9); 8.1 ± 4.2
Neutrophile, /nL	240	5.0 (3.4–7.6); 7.0 ± 12.4
D-Dimer, mg/L	214	1.04 (0.53–2.13); 4.47 ± 14.60
Creatinine, mg/dL	398	1.1 (0.8–1.4); 1.4 ± 1.7
C-reactive protein, mg/L	398	65 (28–121); 87± 81
Lactate dehydrogenase, U/L	284	329 (256–455); 436 ± 832
Activated partial thromboplastin time, s	297	32 (29–37); 35 ± 15
Potassium, mmol/L	395	3.97 (3.62–4.36); 4.03 ± 0.63
Procalcitonin, µg/L	252	0.10 (0.05–0.32); 1.61 ± 11.68
High sensitivity troponin I, ng/L	311	14 (8–37); 537 ± 7022
Hemoglobin, g/dL	400	13.3 (11.7–14.6); 13.1± 2.5
Thrombocytes, /nL	395	208 (160–280); 226 ± 94
International normalized ratio (INR)	336	1.11 (1.03–1.23); 1.24 ± 0.54
Sodium, mmol/L	395	137.0 (134.0–140.0); 137.4 ± 5.9
Aspartate Aminotransferase, U/L	95	48 (32–82); 98 ± 277
Bilirubin, mg/dL	86	0.60 (0.40–0.80); 0.78 ± 0.90
N/L Ratio	217	5.0 (2.9–9.5)
Quick, %	336	84 (71–95); 81 ± 21
Glomerular filtration rate, mL/min	397	65 (43–85); 61 ± 25
Thyroid stimulating hormon, mU/L	303	1.0 (0.6–1.6); 1.6 ± 3.5
Glycated Hemoglobin (HbA1c) (%)	41	6.3 (5.8–8.4); 24.8 ± 98.4
Interleukin 6, pg/mL	22	47 (22–102); 2774 ± 12488
Lactate (mmol/dL)	282	1.4 (1.0–2.0); 3.3 ± 16.1
BE (Base excess), mmol/L	212	1.2 (1.3- 3.2); 1.0 ± 8.3
Creatine Kinase (CK), U/L	333	116 (58–314); 1734 ± 23932
CK-myocardial band (CK-MB), U/L	209	25 (18–41); 40 ± 49
N-terminal prohormone of brain natriuretic peptide (NT-proBNP), ng/L	26	1241 (169–4190); 5052 ± 7735
Troponin, ng/L	311	14 (8–37); 537 ± 7022
**Baseline Electrocardiogram**		
**Rhythm at baseline**		
Sinus rhythm, prop(*n*)		0.84 (286)
Atrial fibrillation, prop(*n*)		0.16 (53)
Heart Rate, /min		88 (77–103); 91 ± 22
ST-segment abnormalities *		0.1 (40)
**Intensive care treatment**		
Need for ventilation, prop(*n*)	414	0.21 (86)
Hours of ventilation	86	354 (168–582); 479± 621
primary non-invasive ventilation, prop(v)	86	0.26 (22)
primary invasive ventilation, prop(*n*)	86	0.74 (64)
progress from non-invasive to invasive ventilation, prop(*n*)	22	0.68 (15)
days of intensive care treatment	116	12 (4–23); 16 ±14
Extracorporeal membrane oxygenation therapy, prop(*n*)	414	0.02 (10)
Invasive coronary angiogram (ICA), prop(*n*)		0.05 (19)
-requiring percutaneous coronary intervention of these with (ICA),prop(*n*)		0.58 (11)

Values are median (first-third quartile), mean ± standard deviation or proportions (*n*). * indicates any form of abnormal ST segments (ST-segment elevation, persistent or transient ST-segment depression, T-wave inversion, flat T waves, or pseudonormalization of T waves).

**Table 2:** Comorbidities and Medication-Hamburg Cohort.

	(*n* = 414)
**Comorbidities**	
Diabetes mellitus, prop(*n*)	0.25 (103)
Hypertension, prop(*n*)	0.55 (226)
Dyslipidemia, prop(*n*)	0.15 (61)
Smoking, prop(*n*)	0.07 (28)
Cardiomyopathy, prop(*n*)	0.05 (20)
Coronary artery disease, prop(*n*)	0.17 (69)
Myocardial infarction, prop(*n*)	0.07 (29)
CABG *, prop(*n*)	0.03 (12)
Prior percutaneous coronary intervention, prop(*n*)	0.07 (31)
Vascular disease ^†^, prop(*n*)	0.21 (87)
Prior arrhythmias, prop(*n*)	0.18 (75)
Implanted device ^‡^, prop(*n*)	0.04 (16)
Congenital heart disease, prop(*n*)	0.01 (5)
Chronic kidney disease, prop(*n*)	0.19 (79)
Chronic liver disease, prop(*n*)	0.02 (9)
Pulmonary disease, prop(*n*)	0.15 (64)
**Medication**	
Antiplatelet therapy, prop(*n*)	0.23 (96)
Oral anticoagulation, prop(*n*)	0.15 (63)
ACE-Inhibitor/ARB ^§^, prop(*n*)	0.37 (155)
Aldosterone Antagonist, prop(*n*)	0.06 (23)
Angiotensin-receptor-neprilysin-inhibito, prop(*n*)	0 (2)
Antidiabetic medication ^II^, prop(*n*)	0.19 (79)
Diuretics, prop(*n*)	0.25 (103)
Statins, prop(*n*)	0.21 (88)
Betablocker, prop(*n*)	0.3 (124)
Antiarrhythmic drugs, prop(*n*)	0.05 (22)
Immuno-suppressive medication ^#^, prop(*n*)	0.08 (33)
Prednisolone, prop(*n*)	0.04 (18)
Proton pump inhibitor, prop(*n*)	0.28 (115)

Values are proportions (*n*). * indicates coronary artery bypass graft. ^†^ vascular disease includes Stroke/transient ischemic attacks, peripheral arterial disease, pulmonary embolism, hemorrhage, aneurysms, left atrial appendage thrombus, thrombosis. ^‡^ Implanted devices include all sorts of pacemakers, implantable cardioverter defibrillators and cardiac resynchronization devices. ^§^ ACE indicates angiotensin-converting-enzyme, ARB indicate angiotensin-receptor-blocker. ^II^ antidiabetic medication includes all oral antidiabetics and/or insulin/insulin analogs. ^#^ Immuno-suppressive medication summarizes all immunosuppressors, targeted therapies, chemotherapeutic agents.

**Table 3:** Primary outcome.

	Hamburg Cohort *n* = 414	Total Cohort *n* = 4704
Primary Endpoints		
At least one cardiovascular event per patient, prop(*n*)	0.22 (92)	0.29 (1364)
Number of total cardiovascular events, *n* (per patient, pp)	132 (1.4 pp)	1845 (1.4 pp)
Cardiopulmonary resuscitation, prop(*n*)	0.03 (12)	0.01 (64)
Cardiogenic shock, prop(*n*)	0.02 (10)	0.01 (28)
Acute coronary syndrome, prop(*n*):	0.03 (11)	0.02 (103)
STEMI *	*0.18 (2)*	*0.19 (20)*
*NSTEMI* ^†^	*0.82 (9)*	*0.81 (83)*
Worsening or new onset heart failure, prop(*n*)	0.1 (43)	0.1 (464)
De novo Arrhythmia (all), prop(*n*):	0.07 (31)	0.20 (927)
*Atrial tachyarrhythmias* ^‡^, prop(*n*)	*0.87 (27)*	*0.90 (837)*
*Other arrhythmias ^§^,* prop(*n*):	*0.13 (4) ^II^*	*0.10 (90)*
- *Supraventricular arrhythmias,* prop(*n*):	*0*	*N/A*
- *Ventricular arrhythmias,* prop(*n*):	*0.13 (4)*	*0.03 (24)*
- *Ventricular tachycardia,* prop(*n*):	*0.06 (2)*	*N/A*
◦ *Ventricular fibrillation,* prop(*n*):	*0.06 (2)*	*N/A*
Acute Myocarditis, prop(*n*)	0.005 (2)	0 (11)
Pulmonary embolism, prop(*n*)	0.03 (11)	0.02 (88)
Thrombosis, prop(*n*)	0.02 (9)	0.02 (78)
Ischemic stroke, prop(*n*)	0.01 (3)	0.02 (82)
Mortality		
Death, prop(*n*)	(90)	0.19 (890)

Values are mean ± standard deviation, median (first-third quartile) or proportions (*n*). N/A indicates not available. * STEMI indicates ST-elevation myocardial infarction. ^†^ NSTEMI indicates non-ST-segment elevation myocardial infarction. ^‡^ atrial tachyarrhythmias includes atrial fibrillation, atrial flutter, atrial tachycardia. ^§^ summarizing all other arrhythmias (supraventricular arrhythmias, ventricular tachycardia and ventricular fibrillation). ^II^ of these four events, there were 2 ventricular tachycardias (one patient with respiratory and cardiac decompensation, occurrence of ventricular tachycardia, CPR and death during hospital stay without any prior heart disease; one patient with respiratory and cardiac decompensation, septic shock, multiple organ failure with ventricular tachycardia and death) and 2 patients with ventricular fibrillation (one patient had VF associated with STEMI and successful resuscitation and percutaneous coronary intervention, discharged to home and one patient with respiratory failure, cardiogenic shock, successful resuscitation of VF, survivor discharged to weaning home).

## Data Availability

All relevant data are within the manuscript and its Supplementary Materials.

## Supplementary Materials

The following are available online at https://www.mdpi.com/article/10.3390/jcm10173982/s1, S1: Listing of all variables from the data set, S2: Definition of cardiovascular events, S3: References regarding the statistical analysis, S4: Incidences of cardiovascular events throughout the different months of the study timespan.
