# Supplementary material for: Prognostic Impact of Acute Cardiovascular Events in COVID-19 Hospitalized Patients—Results from the CORONA Germany Study
Source: J Clin Med. 2021 Sep 2;10(17):3982. doi: 10.3390/jcm10173982 (PMC8432202; doi:10.3390/jcm10173982)
Supplement: Supplementary file 1 [file jcm-10-03982-s001.zip › jcm-1306839-SI.pdf]

Supplement Material to EURHEARTJ-D-20-05457: „ Prognostic impact of acute cardiovascular events in COVID-19 hospitalized patients- - Results from the CORONA Germany study "

S1. Listing of all variables from the data set (Hamburg cohort analysis of six hospitals)

a.	Hospital site
b.	Death (n)
c.	Admission to intensive care unit (n)
d.	Need for mechanical ventilation (n)
e.	Cardiovascular event (n)
**General findings and at admission:**	
1.	Gender (male)
2.	Age (years)
3.	BMI [kg/m²]
4.	Type of admission (walking, rescue service, emergency doctor)
5.	Respiratory rate [/min]
6.	SpO2 [%]
7.	Heart rate [/min]
8.	Systolic blood pressure [mmHg]
9.	Diastolic blood pressure [mmHg]
10.	Duration of symptoms onset until hospitalization
11.	Sick contact (n)
12.	First allocation (normal ward, intermediate care IMC) or ICU)
13.	Worsening of clinical condition (upgrade to IMC/ICU)
14.	Headache (n)
15.	Diarrhea (n)
16.	Glasgow coma scale < 15 (n)
17.	Expectoration (n)
18.	Sore throat (n)
19.	Loss of smell/taste (n)
20.	Body aches (n)
21.	Nausea (n)
22.	Edema (n)
23.	Chills (n)
24.	Body temperature [°C]
25.	Fever (>38.5°C) (n)
26.	Dyspnea (n)
27.	Cough (n)
28.	Baseline ECG: Rhythm (sinus rhythm/ atrial fibrillation/ supraventricular tachycardia/ atrial tachycardia/ atrial flutter/ ventricular tachycardia and ventricular fibrillation)
29.	Baseline ECG: Heart rate [/min]
30.	Baseline ECG: ST segment abnormalities (n)
**Baseline laboratory findings:**	
31.	Hemoglobin [g/dl]
32.	Leukocytes [/nl]
33.	Thrombocytes [/nl]
34.	Neutrophils [/nl]
35.	Lymphocytes [/nl]
36.	N/L Ratio
37.	Activated partial thrombin time [seconds]

38. INR
39. D-Dimer [mg/l]
40. Creatinine [mg/dl]
41. Potassium [mmol/l]
42. Sodium [mmol/l]
43. C-reactive protein [mg/l]
44. Lactate dehydrogenase [U/l]
45. Aspartate aminotransferase, U/l
46. Bilirubin, mg/dl
47. Creatine Kinase (CK), U/l
48. CK-myocardial band (CK-MB), U/l
49. N-terminal prohormone of brain natriuretic peptide (NT-proBNP), ng/l
50. High sensitivity troponin I, ng/l
51. Procalcitonin, µg/l
52. Interleukin 6, pg/ml
53. Lactate (mmol/dl)
54. BE (Base excess)
55. Quick
56. Fibrinogen, g/L
57. Glomerular filtration rate [ml/min]
58. Thyroid-stimulating hormone, TSH, mU/l
59. HbA1c (%)
**Prior comorbidities:**
60. Diabetes mellitus (n)
61. Hypertension (n)
62. Cardiomyopathy (n)
63. Coronary artery disease (n)
64. Myocardial infarction (n)
65. AC(V)B (Bypass) (n)
66. Prior arrhythmias (n)
67. Cardia device, (n)
68. Vascular disease (n)
69. Dyslipidemia (n) (including hypertriglyceridemia, hypercholesterolemia, combined or hyperlipoproteinemia)
70. Pulmonary disease (n)
71. Chronic kidney disease (n)
72. Chronic liver disease (n)
73. Cancer (n)
74. Congenital heart disease (n)
75. Prior percutaneous coronary intervention (n)
76. Ongoing tumor disease (n)
77. Metastasis (n)
78. Prior NYHA classification (I-IV)
79. Prior LV-function (n)
80. Smoking (n)
81. Prior organ transplantation (n)
**Prior medication:**
82. Antiplatelet medication (n)
83. Oral anticoagulation (n)
84. Betablocker (n)
85. Antiarrhythmic drug (n)
86. Renin-Angiotensin-System Blocker
87. Aldosterone Antagonist (n)
88. Statins (n)
89. Diuretics (n)

90.	Angiotensin-Receptor-Neprilysin-Inhibitor (n)
91.	Antidiabetic medication, oral and/or insulin (n)
92.	Immuno-suppressive medication including prednisolon, targeted therapies and immunosuppressors (n)
93.	Prednisolone intake (n)
94.	Proton pump inhibitor (n)
**Maximum value:**	
95.	Maximum high sensitivity troponin I, ng/l

S2. Definition of cardiovascular events

Cardiovascular event	Definition	Literature Reference
**1) Cardiopulmonal Resuscitation (CPR) in cardiac arrest**	Confirmed cardiac arrest with start of CPR (including compression, ventilation and if applicable defibrillation)	Monsieurs KRG, Nolan JP, Bossaert LL, Greif R, Maconochie IK, Nikolaou NI,et al. European Resuscitation Council Guidelines for Resuscitation 2015. Section 1. Executive summary. Resuscitation. 2015;95:1–80.
**2) Cardiogenic Shock**	Cardiogenic shock was defined as hypotension (SBP <90 mmHg) despite adequate filling status with signs of hypoperfusion. There had to be evidence of peripheral vasoconstriction such as oliguria, cyanosis and/or diaphoresis.	Ponikowski P, Voors AA, Anker SD, Bueno H, Cleland JGF, Coats AJS, et al. 2016 ESC Guidelines for the diagnosis and treatment of acute and chronic heart failure. Eur Heart J. 2016;37(27):2129–2200m.
**3) Acute coronary syndrome (ACS) including NSTEMI, STEMI**	A combination of criteria is required to meet the diagnosis of AMI, namely the detection of an increase and/or decrease of a cardiac biomarker, preferably high-sensitivity cardiac troponin (hs-cTn) T or I, with at least one value above the 99th percentile of the upper reference limit and at least one of the following:<br>(1) Symptoms of myocardial ischemia.<br>(2) New ischemic ECG changes.<br>(3) Development of pathological Q-waves on ECG.<br>(4) Imaging evidence of loss of viable myocardium or new regional wall motion abnormality in a pattern consistent with an ischemic etiology.<br>(5) Intracoronary thrombus detected on angiography or autopsy.<br>Patients with acute chest pain and persistent (>20 min)<br>ST-segment elevation: this condition is termed ST-segment elevation	Thygesen K, Alpert JS, Jaffe AS, Chaitman BR, Bax JJ, Morrow DA, et al. Fourth Universal Definition of Myocardial Infarction (2018). Vol. 138, Circulation. 2018. 618-651 p.<br><br>Collet J-P, Thiele H, Barbato E, Barthélémy O, Bauersachs J, et al. 2020 ESC Guidelines for the management of acute coronary syndromes in patients presenting without persistent ST-segment elevation. #Eur Heart J. 2020;1–79.

		ACS and generally reflects an acute total or subtotal coronary occlusion. Most patients will ultimately develop ST-segment elevation myocardial infarction (STEMI). Patients with acute chest discomfort but no persistent ST-segment elevation [non-ST-segment elevation ACS (NSTEACS)] exhibit ECG changes that may include transient ST-segment elevation, persistent or transient ST-segment depression, T-wave inversion, flat T waves, or pseudonormalization of T waves; or the ECG may be normal.	
**4)**	**Acute myocarditis**	Diagnosed either by endomyocardial biopsy or by cardiac magnetic resonance imaging (which is the non- invasive gold-standard method for the diagnosis of myocarditis)	Caforio ALP, Pankuweit S, Arbustini E, Basso C, Gimeno-Blanes J, Felix SB, et al. Current state of knowledge on aetiology, diagnosis, management, and therapy of myocarditis: A position statement of the European Society of Cardiology Working Group on Myocardial and Pericardial Diseases. Eur Heart J. 2013;34(33):2636–48<br><br>T schöpe C, Ammirati E, Bozkurt B, Caforio ALP, Cooper LT, Felix SB, et al. Myocarditis and inflammatory cardiomyopathy: current evidence and future directions. Nat Rev Cardiol. 2020;
**5)**	**De novo cardiac arrhythmia**	Detection of a new arrhythmia on an ECG for at least 30 seconds during current hospitalization without prior history of documented cardiac arrhythmias	Hindricks G, Potpara T, Serbia C, Germany ND, Arbelo E, Blomstro C, et al. 2020 ESCGuidelines for the diagnosis and management of atrial fibrillation developed in collaboration with the EuropeanAssociation of Cardio-Thoracic Surgery (EACTS). Eur Heart J. 2020;1–126.<br><br>Brugada J, Katritsis DG, Arbelo E, Arribas F, Bax JJ, Blomstrom-Lundqvist C, et al. 2019 ESC Guidelines for themanagement of patients with supraventricular tachycardia. Eur Heart J. 2020;41(5):655–720.<br><br>Priori SG, Blomstrom-Lundqvist C, Mazzanti A, Bloma N, Borggrefe M, Camm J, et al. 2015 ESC Guidelines for the management of patients with ventricular arrhythmias and the prevention of sudden cardiac death the Task Force for the Management of Patients with Ventricular Arrhythmias and the Prevention of Sudden Cardiac Death of the European Society of Cardiology (ESC) Endorsed by: Association for European Paediatric and Congenital Cardiology (AEPC). Eur Heart J. 2015;36(41):2793–2867l.
**6)**	**Thrombosis (Arterial thromboembolism or venous)**	Clinical picture of arterial TE (signs for organ hypoperfusion, acute ischemia) and confirmed by imaging (echocardiography, computer tomography (CT)) or venous TE (e.g. swelling, overheated extremity, pain); diagnosed by imaging (duplex/CT/etc.)	Konstantinides S V., Meyer G, Bueno H, Galié N, Gibbs JSR, Ageno W, et al. 2019 ESC Guidelines for the diagnosis and management of acute pulmonary embolism developed in collaboration with the European respiratory society (ERS). Eur Heart J. 2020;41(4):543–603.

**7) Pulmonary artery embolism (PAE)**	Confirmed PAE by imaging (including Computed tomographic pulmonary Angiography, ventilation/perfusion lung scintigraphy or single-photon emission computed tomography; pulmonary angiography	Konstantinides S V., Meyer G, Bueno H, Galié N, Gibbs JSR, Ageno W, et al. 2019 ESC Guidelines for the diagnosis and management of acute pulmonary embolism developed in collaboration with the European respiratory society (ERS). Eur Heart J. 2020;41(4):543–603.
**8) Worsening of prior or new heart failure**	rapid onset or worsening of symptoms and/or signs of heart failure, including signs of congestion and/or hypoperfusion. Clinical signs of right-/and or left heart failure with pulmonary congestion, orthopnea/edema/pleura effusion, peripheral edema, jugular venous dilatation, congested hepatomegaly, gut congestion (ascites), hepatojugular reflux; cold sweated extremities, oliguria, mental confusion, dizziness, narrow pulse pressure and supportive findings on ECG or chest radiograph<br>- Including New York Heart Association class III and IV	Ponikowski P, Voors AA, Anker SD, Bueno H, Cleland JGF, Coats AJS, et al. 2016 ESC Guidelines for the diagnosis and treatment of acute and chronic heart failure. Eur Heart J. 2016;37(27):2129–2200
**9) Ischemic stroke**	Diagnosed by Non-contrast brain computer tomography or brain magnetic resonance tomography	Jauch EC, Saver JL, Adams HP, Bruno A, Connors JJB, Demaerschalk BM, et al. Guidelines for the early management of patients with acute ischemic stroke: A guideline for healthcare professionals from the American Heart Association/American Stroke Association. Stroke. 2013;44(3):870–947

S3. References regarding the statistical analysis

1. Allaire, JJ, Yihui Xie, Jonathan McPherson, Javier Luraschi, Kevin Ushey, Aron Atkins, Hadley Wickham, Joe Cheng, Winston Chang, and Richard Iannone. 2019. *Rmarkdown: Dynamic Documents for R*. https://rmarkdown.rstudio.com.

2. Francois, Romain. 2017. *Bibtex: Bibtex Parser*. https://CRAN.R-project.org/package=bibtex.

3. Harrell Jr, Frank E, with contributions from Charles Dupont, and many others. 2020. *Hmisc: Harrell Miscellaneous*. https://CRAN.R-project.org/package=Hmisc.

4. R Core Team. 2020. *R: A Language and Environment for Statistical Computing*. Vienna, Austria: R Foundation for Statistical Computing. https://www.R-project.org/.

5. Xie, Yihui, J. J. Allaire, and Garrett Grolemund. 2018. *R Markdown: The Definitive Guide*. Boca Raton, Florida: Chapman; Hall/CRC. https://bookdown.org/yihui/rmarkdown.

S4. Incidences of cardiovascular events throughout the different months of the study timespan

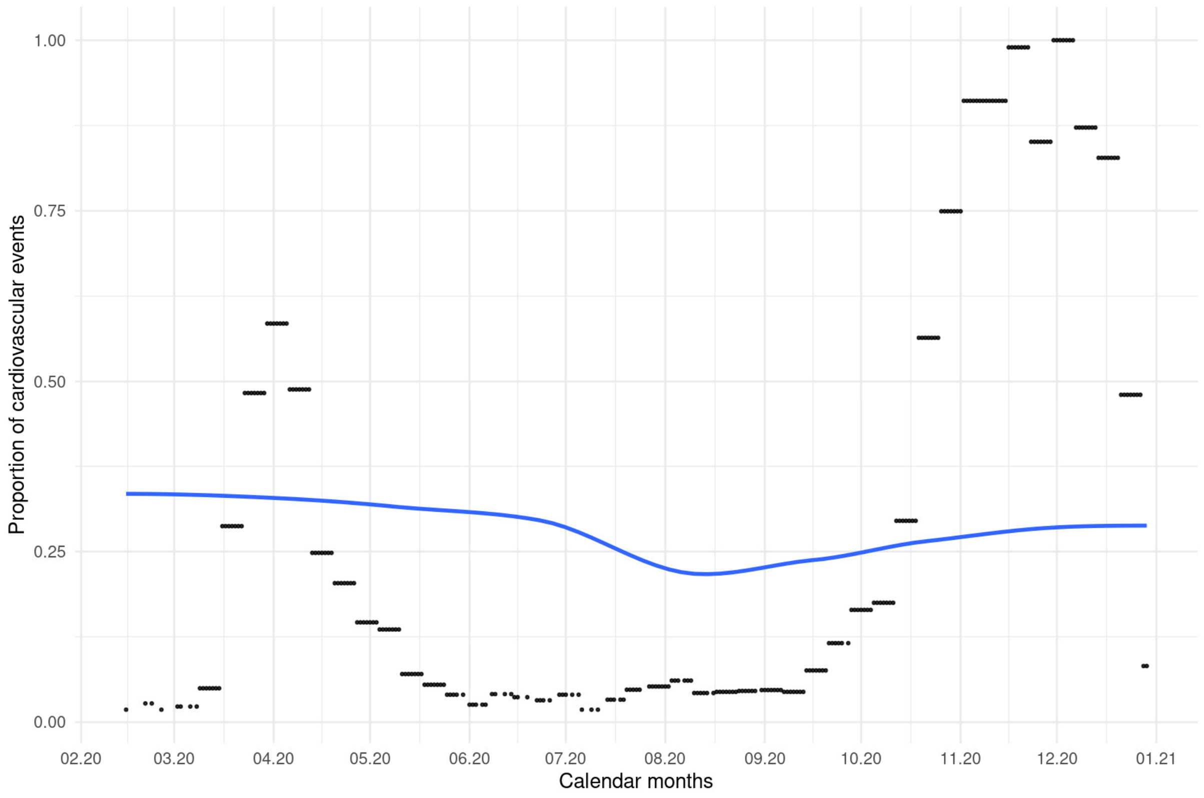

Black points represent CV events in COVID-19 cases within observational period scaled to maximum == 1.
